# Isolation, identification, and application of lactic acid-producing bacteria using salted cheese whey substrate and immobilized cells technology

**DOI:** 10.1186/s43141-022-00316-5

**Published:** 2022-02-11

**Authors:** Atiat Sayed Dosuky, Tarek Ragab Elsayed, Eman Tawfik Yousef, Olfat Sayed Barakat, Nasr Fawzy Nasr

**Affiliations:** 1 Food Technology Research Institute, Agricultural Research Center, Giza, 12619 Egypt; 2 Agricultural Microbiology Department, Faculty of Agriculture, Cairo University, Giza, 12613 Egypt

**Keywords:** Immobilization, Lactic acid bacteria, Salted cheese whey, Whey permeate

## Abstract

**Background:**

Lactic acid bacteria (LAB) could be used for bio-production of lactic acid (LA) from wastes of dairy industries. This study aimed to produce LA using isolated and identified LAB capable of withstanding high salt concentration of salted cheese whey and adopting immobilization technique in repeated batch fermentation process.

**Results:**

Seventy four isolates of LAB were isolated from salted cheese whey and examined for lactic acid production. The superior isolates were biochemically and molecularly identified as *Enterococcus faecalis*, *Enterococcus faecium*, and *Enterococcus hirae*. Then the best of them, *Enterococcus faecalis*, *Enterococcus hirae* and dual of them besides *Lacticaseibacillus casei* were immobilized by sodium alginate 2% in entrapped cells. Repeated batch fermentation was executed for LA production from the mixture of salted whey and whey permeate (1:1) using immobilized strains during static state fermentation under optimum conditions (4% inoculum size in mixture contained 5% sucrose and 0.5% calcium carbonate and incubation at 37 °C). The potent bacterial strain was *Enterococcus faecalis* which gave the maximum LA production of 36.95 g/l with a yield of 81% after 36 h incubation at 37 °C in presence of 5% sugar.

**Conclusion:**

Immobilized cells exhibited good mechanical strength during repetitive fermentations and could be used in repetitive batch cultures for more than 126 days.

## Background

Lactic acid (LA) is widely used for various applications in food and non-food industries. It has served as a raw material in the production of valuable products [1]. The commercial production level of LA can be achieved using either chemical synthesis or microbial fermentation. However, fermentation method is more preferable due to environment and substrate concerns related to chemical method [2].

Lactic acid bacteria (LAB) are classified into two groups; homofermentative and heterofermentative. Homofermentative LAB can exclusively produce only lactic acid from glucose, while heterofermentative LAB produce other substances like ethanol and CO_2_ with LA from glucose. LAB typically needs complex nutritional requirements for growth, because of their limited ability to synthesize their own growth factors such as B vitamins and amino acids. They need some elements for growth such as carbon and nitrogen sources in form of carbohydrates, amino acids, vitamins, and minerals [3].

Cheese whey is an industrial waste or by-product of dairy plants. It could be used as a substrate for lactic acid production. Whey contains lactose, protein, fat, and mineral salts. Microorganisms such as *Lactobacillus helveticus* [4] and *Lacticaseibacillus casei* [5] are used for production of lactic acid from cheese whey. Whey permeate is a by-product obtained when whey is passed through an ultrafiltration membrane to concentrate milk proteins, so whey proteins are retained by the membrane, whereas smaller molecules such as lactose and salts pass through the membrane forming the whey permeate. Whey permeate is readily a valuable and low cost substrate for production of lactic acid [6].

Immobilization of microbial cells is among the procedures to increase cell retention and cell density in the bioreactors, also immobilization of cells provides minimal lag phase, tolerance to high concentration of sugar, improvement of pH control, and high productivity. Several materials such as Ca-alginate gels, polyethylene amine and plastic composite support have been utilized for immobilization of LAB cells to produce lactic acid [7, 8].

The aims of the present work were isolation and identification of lactic acid bacteria from salted cheese whey, maximizing lactic acid production from a mixture of salted cheese whey and whey permeate using free and immobilized cells of different LAB isolates using repeated batch culture technique.

## Methods

### Microorganisms

*Lacticaseibacillus casei* was obtained from department of Microbiology in a faculty of Agriculture, to examine its potential to produce lactic acid as a reference strain. The strain was stored at − 20 °C by mixing the fresh sub-cultures with 20% glycerol [9]. Before testing, the strain was sub-cultured at appropriate temperature in sterile MRS agar medium.

### Raw materials

Salted cheese whey (contained total solids 10.5%; lactose 4.9%, protein 0.8%, fat 0.12%, salt 4%, and ash 0.09%) was obtained from Dairy Science department in a faculty of Agriculture and used for isolation of LAB. Whey permeate (5% lactose) was obtained from Dairy Industry Unit in a research center. Chemical composition of raw materials was determined according to [10]. Salted whey and whey permeate were deproteinized by heating as described by [11].

### Isolation of LAB from salted cheese whey

A sample of salted cheese whey was applied using plate count method on selective agar media, the M17 agar medium for lactococci and enteroccoci [12], MRS medium for lactobacilli [13]. Developed typical colonies were picked up and purified twice. Pure cultures were grown on M17 or MRS agar at 37 °C for 24 h and stored at − 20 °C with 20% glycerol [9]. Before using the isolates, frozen cultures were sub-cultured overnight.

### Morphological and biochemical characterization of LAB isolates

Isolates were cultivated on M17 or MRS agar media at 37 °C for 24 h and used for identification and fermentation process. LAB were phenotypically identified based on morphological and biochemical characters; Gram staining, oxygen requirements, catalase activity [14], litmus milk test [15], CO_2_ production from glucose and fermentation of sugars (sucrose, mannitol, rhamnose, sorbitol, and maltose), as well as growth at 10, 45, and 50 °C in 6.5% NaCl and pH 9.6 according to [16], Phenol red broth base medium [17] is recommended for carbohydrate fermentation. Growth of bacterial isolates at deferent temperatures 10, 45, and 50 °C in 6.5% NaCl and pH 9.6 was tested in M17 or MRS*.*

### Screening of isolates for lactic acid production

Different set of batches were carried out to study the ability of bacterial isolates to produce lactic acid using different fermentation media containing (salted cheese whey, whey permeate, as well as mixture of both (1:1)) supplemented with SN nutrients (manganese sulphate 20 mg/l, yeast extract 0.75%) at temperature 37 °C for 36 h using inoculum size 4%. All batches were carried out in conical flasks containing 100 ml working volume at static state fermentation.

### Molecular identification of efficient LA-producing isolates

#### Bacterial DNA extraction

Bacterial isolates were grown in broth medium for 24 h at 37 °C then harvested by centrifugation at 12,000 g for 5 min. After washing of bacterial pellets for three times using 0.85% NaCl saline, genomic deoxyribonucleic acid (DNA) was extracted using Gene JET genomic DNA purification Kit (Thermo scientific, Lithuania) [18]. DNA yields and purity were checked using both nanodrop spectrophotometer and agarose gel electrophoresis.

#### Bacterial fingerprints and genotypic diversity

The BOX-PCR fingerprints of bacteria were generated according to [19] using BOXA1R primer (CTACGGCAAGGCGACGCTGACG). Eight microliters of the PCR products were separated by 1.5% agarose gel electrophoresis in 0.5 X TBE-buffer for 4 h (50 V). The BOX-PCR fingerprints patterns were checked and compared visually.

#### Identification of bacterial isolates by 16S rRNA gene sequencing

The 16S ribosomal ribonucleic acid (rRNA) gene fragments of 6 lactic acid-producing isolates were amplified using the universal primers F-27 (5′-AGAGTTTGATCMTGGCTCAG-3′) and R1494 (5′-CTACGGYTACCTTGTTACGAC-3′) using PCR machine (Bio-rad T100 thermal cycler). PCR products were checked via agarose gel electrophoresis then purified using gel extraction kit (Thermo scientific, Lithuania) and sequenced by Macrogen, Koria.

#### Phylogenic analysis of bacterial isolates

The evolutionary history was inferred using the neighbor-joining method. The tree was computed using the maximum composite likelihood method. The analysis involved 28 nucleotide sequences of which 6 sequences of 16S rRNA gene amplified from bacterial isolates of current study while 22 sequences representing the most similar hits were obtained from the National Center for Biotechnology Information (NCBI) gene bank data base. Evolutionary analyses were conducted in MEGA5 software.

### Optimization of parameters for LA production using immobilized cells

Optimization of process parameters for LA production using the most efficient two bacterial isolates (Ent.58 and Ent.68) and *L*. *casei* were carried out in batches in 250 ml conical flask containing the fermentation media (100 ml working volume of whey permeate and salted cheese whey (1:1)) using immobilized cells and repeated batches technique to study the effects of calcium carbonate (CaCO_3_, 0.5%), incubation temperature (30, 37, and 45 °C), inoculum size (2–4%), and different concentrations of sucrose (5 and 10%) during fermentation for 36 h under static state fermentation conditions. Bacterial cultures were examined in M17and MRS synthetic broth media for biomass production, respectively. Conical flasks (250 ml) containing 100 ml of media were inoculated with freshly activated 4% (v/v) inoculum and incubated at 37 °C for 24 h.

### Immobilization and cell entrapment method of bacterial cells

According to [20], immobilization of bacterial isolates (Ent.58, Ent.68, and dual of them) and *L*. *casei* was conducted as follows; cells were harvested by centrifugation at 4000 rpm for 15 min at 4 °C and washed by 0.1% (w/v) sterile water peptone. The pellets were suspended in 5 ml of 0.1% (w/v) sterile water peptone and mixed with equal volume of sodium alginate solution to yield a final alginate concentration of 2%. The mixture was gently added drop-wisely to sterile stirred 1% CaCl_2_ through a needle, where alginate drops have solidified by CaCl_2_ forming beads which entrapped bacterial cells. After 30 min of jellification, beads in diameter 2 mm were washed twice with sterile saline solution to remove un-immobilized cells and excess calcium ions. Then, the beads were rinsed with 0.1% sterile water peptone and stored at 4 °C. Analytical growth of beads was carried out to determine the count of immobilized cells in one gram beads.

### Free and immobilized cells counting

Count of immobilized cells was enumerated; beads (0.1 g) were liquefied in 100 ml of 1% sterilized sodium citrate solution (pH 6.0) and serially diluted in 0.1% water peptone [21]. Dilutions of free and immobilized cells were transferred into plates and counts were determined using M17 or MRS agar according to [13]. The plates were incubated at 37 °C for 48 h under anaerobic conditions [22].

### Repeated batches using immobilized cells for LA production

To find out the optimal incubation time for the maximal lactic acid production, the fermentative medium was inoculated with immobilized bacterial cultures (Ent.58 and Ent.68 and dual of them as well as *L*. *casei*) and incubated for 24, 36, 72, and 96 h. Repeated batch (semi continuous) fermentation in 250 ml conical flasks with working volume of 100 ml were used. Inoculum size of 4% was added to each flask containing 100 ml of salted cheese whey and whey permeate mixture supplemented with SN and incubated at 37 °C for 18, 27, 54, and 72 days representing 18 runs (each run 24, 36, 72, or 96 h, in which 100 ml of fermentation medium were added and 100 ml fermentation culture were withdrawn). At the end of each period, lactic acid production was estimated according to the higher production of LA, as well as LA yield and efficiency (conversion ratio) were calculated as follows:

$$\mathrm{Lactic}\ \mathrm{acid}\ \mathrm{yield}\ \left(\%\right)=\frac{\mathrm{Lactic}\ \mathrm{acid}\ \mathrm{production}}{\mathrm{Sugar}\ \mathrm{utilized}}\times 100$$

$$\mathrm{Conversion}\ \mathrm{ratio}\ \left(\%\right)=\frac{\mathrm{Initial}\ \mathrm{sugar}\ \mathrm{conc}.-\mathrm{residual}\ \mathrm{sugar}\ \mathrm{conc}.}{\mathrm{Initial}\ \mathrm{sugar}\ \mathrm{conc}.}\times 100$$

### Analytical methods

Lactose was measured by an enzymatic method according to [23]. The sample was diluted in distilled water (1: 100), 80 μl were placed in spectrophotometer cuvette, then 20 μl of distilled water, 200 μl of citrate buffer, and 50 μl of lactozyme were added. The mixture was shaken and incubated at 25 °C for 20 min for lactose hydrolysis. Then Peridochrom (2 ml) was added and incubated at 25 °C for 50 min, the developed color was measured at 510 nm in spectrophotometer, and blank solution without lactose was used as well. Reducing sugar was measured using 3,5-dinitrosalicylic acid (DNS) method of [24] as g/l, DNS reagent contains (DNS 1%, phenol 0.2%, sodium sulfite 0.05%, sodium hydroxide 1% and Rochelle salt; sodium potassium tartrate 20%). Three milliliters of DNS reagent were added to 2 ml of aliquot sample in a test tube; the mixture was heated in a boiling water bath for 5 min then cooled to room temperature. Light absorbance of sample and reagent blank was determined using spectrophotometer at 640 nm. Lactic acid was determined using high performance liquid chromatograph (HPLC), before HPLC, samples were filtered using 0.20 μm membrane filters. A Bio-Rad Aminex HPX-87H column (300 × 7.8 mm) packed with a sulphonated divinyl benzene-styrene copolymer was used for the separation of compounds. The mobile phase (0.005 M H_2_SO_4_) was fed at a flow rate of 0.6 ml/min and temperature was kept at 50 °C. After HPLC, lactate concentration was spectrophotometrically estimated using an enzymatic kit (lactate-liquizyme, Schiffgraben, Hannover, Germany); 1 ml of reagent and 10 μl of sample were mixed and incubated at 37 °C for 5 min, then light absorbance was measured at 546 nm wave length for sample and standard blank reagent (lactate conc. = (absorbance of sample / absorbance of standard) × 10) [25].

### Statistical analysis

All experiments were achieved in triplicates in a completely randomized design. The significance of the main factors was estimated by analysis of variance (ANOVA). The significance of variance treatments was assessed by Duncan’s multiple range test (*P* < 0.05). Analyses were estimated using a software package “Costat,” a product of Cohort Software Inc., Berkeley, California. All results were reported as means of three replications. Standard deviation (SD) and least significant difference (LSD) were calculated.

## Results

### Isolation and characterization of LAB from salted cheese whey

Seventy-four isolates of LAB were isolated from salted cheese whey. All the 74 isolates were characterized as Gram positive, catalase negative, non-spore forming, rods, or cocci shaped. Morphological and biochemical results of the isolates are shown in Table 1. In general, the isolates were divided into three genera: *Lactococcus* (31 isolates, 41.89%), *Enterococcus* (30 isolates, 40.54%), and *Lactobacillus* (13 isolates, 17.57%).

**Table 1:** Morphological and biochemical characteristics of lactic acid-producing isolates

Characters	Lactobacilli	Lactococci	Enterococci
Cell shape	Rods	Cocci	Cocci
Oxygen requirements	microaerophilic	facultative	facultative
Gram staining	+	+	+
Catalase test	−	−	−
Growth at 10 °C	−	+	+
Growth at 45 °C	+	−	+
Growth at 50 °C	+	−	+
Growth in 6.5 % NaCl	−	−	+
Growth at pH 9.6	−	−	+
Sugar fermentation			
Sucrose	+	±	+
Mannitol	+	±	+
Maltose	+	+	+
Rhamnose	−	−	± or −
Fructose	+	±	+
Galactose	+	±	+
Xylose	−	+	± or −

### Screening of lactic acid-producing isolates using salted cheese whey and whey permeate mixture (1:1)

The isolates were examined for production of LA in mixture of salted cheese whey and whey permeate (1:1) supplemented with SN at temperature 37 °C for 36 h and inoculum size of 4%. Isolates were sorted according to production of LA (g/l) as follow: 15 isolates produced 15.01 to 16.27 g/l, 29 isolates produced 14.01 to 14.98 g/l, 18 isolates produced 13.03 to 13.99 g/l, and 12 isolates produced 10.95 to 12.87 g/l.

### Production of lactic acid using salted cheese whey

**Fig. 1:**
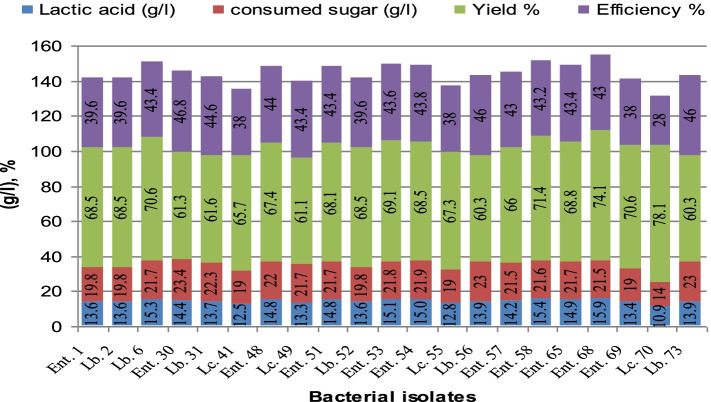
Production of lactic acid using the best 21 isolates in salted whey (LSD 0.05% = 0.381 for isolates)

The superior 21 LA-producing isolates which gave highest LA production levels in salted cheese whey and whey permeate mixture were chosen to study effect of salt on LA production using salted whey (4% salt). Isolates *Ent*.68, *Ent*.58, and *L*.6 gave LA yield ranging between 74.1 and 70.6 %. The highest production of LA was 15.93 g/l with yield 74.1% by isolate *Ent*.68 with efficiency of 43% in Fig. 1.

### Production of lactic acid using whey permeate

The potent ten LA-producing isolates were used to produce LA using different media (whey permeate, salted cheese whey, and mixture of both (1:1)) supplemented with SN at temperature of 37 °C for 36 h and 4% inoculum size. Results in Table 2 showed that when using whey permeate, isolate (*Ent*.68) gave the highest LA production (14.8 ± 0.09 g/l) with yield of 57.4% followed by isolate (*Ent*.58) producing 14.7 ± 0.1 g/l LA with 57% yield. LSD_0.05_ was 0.083 for isolated strains while LSD_0.05_ was 0.046 for culture media. In salted cheese whey medium, the best bacterial isolates were *Ent*.68 and *Ent*.58, which produced LA 15.93 ± 0.13 g/l and 15.43 ± 0.03 g/l with yields of 74.1 and 71.4%, respectively. In whey permeate and salted cheese whey mixture (1:1), *Ent*.68 and *Ent*.58 produced LA 16.27 ± 0.09 g/l and 16.23 ± 0.03 g/l with yields of 55.2 and 56.6%, respectively. Therefore, isolates *Ent*.68 and *Ent*.58 were used for further experiments.

**Table 2:** Lactic acid production using whey permeate, salted whey and their mixture

Whey permeate					Salted whey				Whey permeate: salted whey (1:1)			
Isolates	Lactic acid (g/l) (mean ± SD)	Consumed sugar(g/l)	Yield (%)	Efficiency (%)	Lactic acid (g/l) (mean ± SD)	Consumed sugar(g/l)	Yield (%)	Efficiency (%)	Lactic acid (g/l) (mean ± SD)	Consumed sugar (g/l)	Yield (%)	Efficiency (%)
L. 6	14.66 ± 0.06	26.1	56	52.2	14.35 ± 0.1	23.4	61.3	46.8	15.34 ± 0.05	28.8	53	57.6
Ent. 30	14.04 ± 0.05	26.9	52	53.8	15.31 ± 0.03	21.7	70.6	43.4	16.04 ± 0.04	28.2	57	56.4
Ent. 48	13.26 ± 0.06	22.7	58	45.4	14.83 ± 0.08	22	67.4	44	15.64 ± 0.05	41.6	38	83.2
Ent. 51	10.62 ± 0.12	17	62	34	14.78 ± 0.08	21.7	68.1	43.4	15.45 ± 0.07	41.1	38	82.2
Ent. 53	14.19 ± 0.19	27.5	52	55	15.06 ± 0.06	21.8	69.1	43.6	16.02 ± 0.04	21.6	74	43.2
Ent. 54	13.1 ± 0.11	21.6	61	43.2	15.01 ± 0.08	21.9	68.5	43.8	15.73 ± 0.05	28.6	55	57.2
Ent. 57	13.73 ± 0.13	23.3	59	46.6	14.18 ± 0.16	21.5	66	43	15.31 ± 0.11	39.7	54	79.4
Ent. 58	14.7 ± 0.1	25.8	57	51.6	15.43 ± 0.03	21.6	71.4	43.2	16.23 ± 0.03	28.3	57	56.6
Ent. 65	13.73 ± 0.04	23.3	59	46.6	14.92 ± 0.02	21.7	68.8	43.4	15.7 ± 0.11	28.1	56	56.2
Ent. 68	14.8 ± 0.09	25.8	57.4	51.6	15.93 ± 0.13	21.5	74.1	43	16.27 ± 0.09	27.6	59	55.2

Initial sugar concentration, 5%; CaCO_3,_ 0.5; L., *Lactobacillus*; Ent., *Enterococcus*

LSD_0.05_= 0.083 for isolates, LSD_0.05_= 0.046 for culture media

### Molecular identification of efficient isolates for LA production

#### Bacterial fingerprints and genotypic diversity

**Fig. 2:**
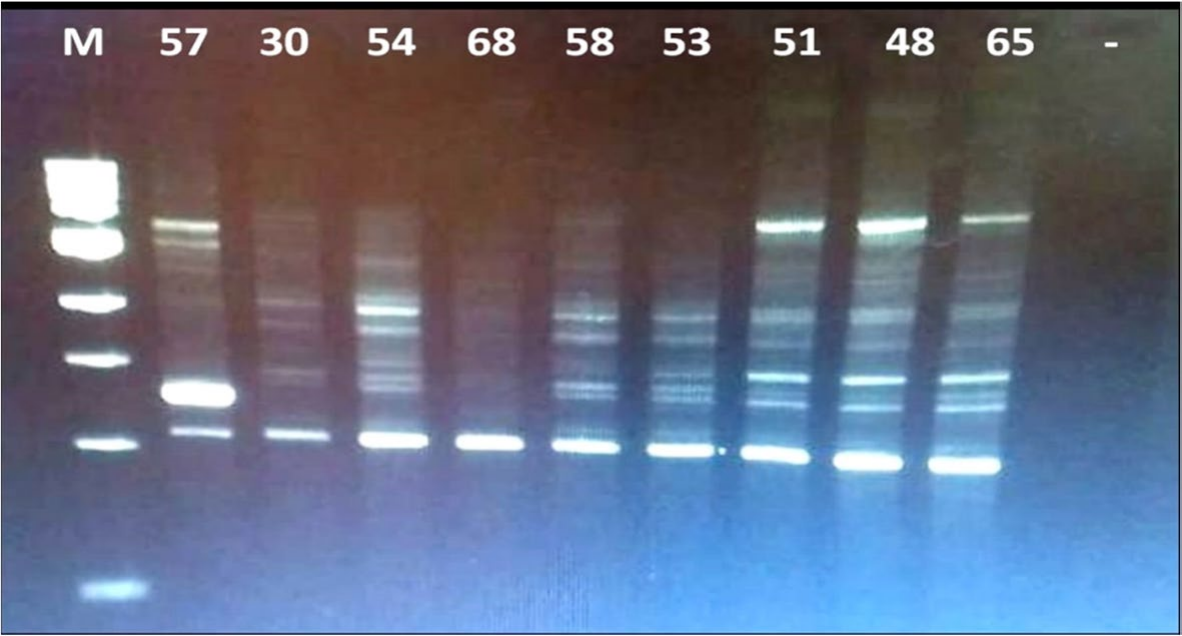
BOX-PCR fingerprints of 9 bacterial isolates obtained from salted whey; M, 1Kb ladder

**Fig. 3:**
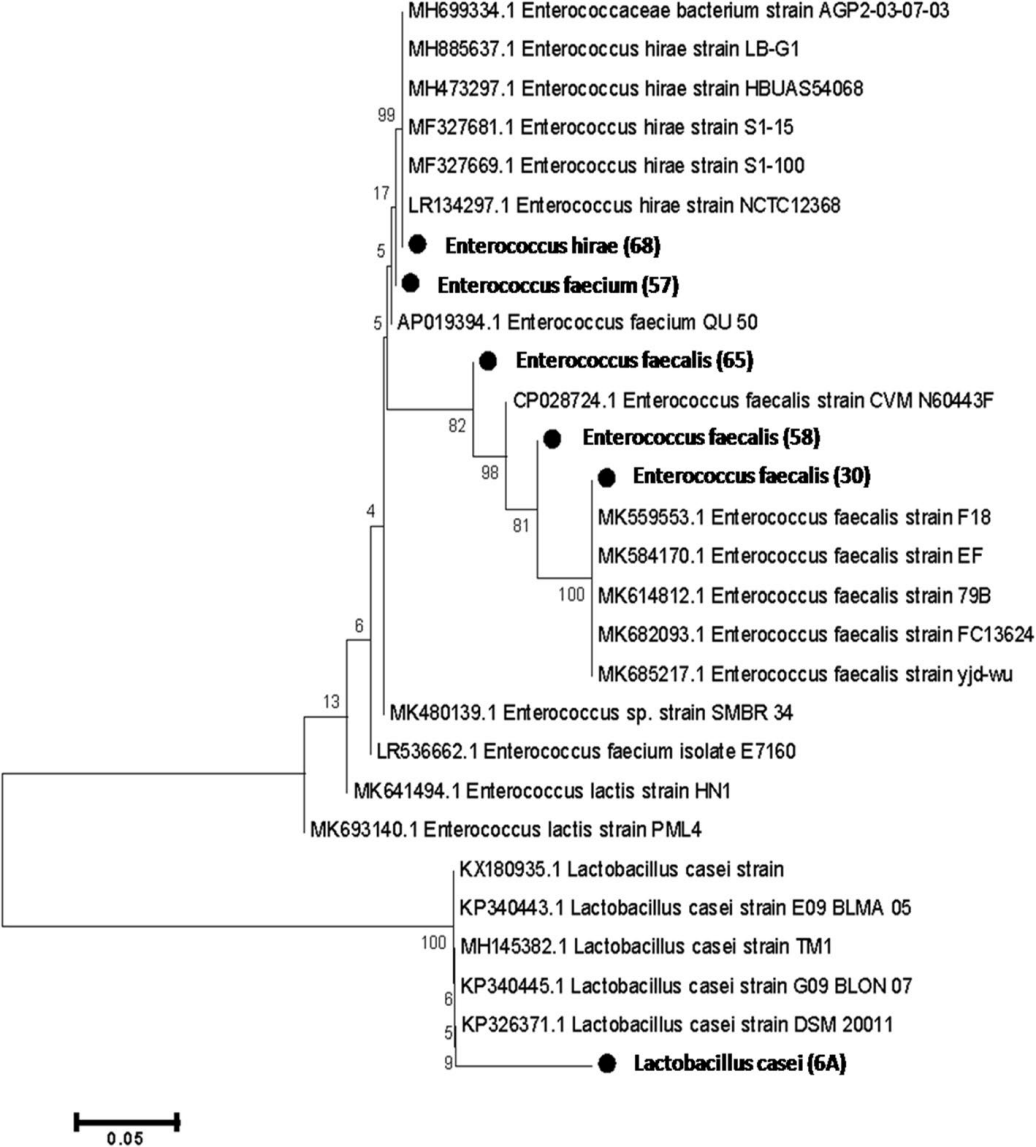
A neighbor-joining phylogenetic tree based on 16S rRNA gene sequences of 9 LAB isolates

BOX-PCR fingerprints were generated for 9 bacterial isolates obtained from salted cheese whey. The fingerprint profiles in Fig. 2 showed the genotypic diversity of tested isolates, identical fingerprint profiles were detected among the isolates 48, 51, and 65 and also among the isolates 53, 54, and 58. One representative isolate of each different fingerprint profile was identified based on the sequence of 16S rRNA gene. Furthermore, the fingerprint profiles confirmed by the results of 16S rRNA gene sequencing, as more similar fingerprint profiles (isolates; 30 and 58) compared to the fingerprint profile of isolate 65, showed also higher similarity based on 16S rRNA gene sequences as shown in the phylogenetic tree in Fig. 3. Dark circles refer to the closest hits obtained from the NCBI gene bank.

#### Identification of bacterial isolates by 16S rRNA gene sequencing

The 16S rRNA gene sequence analysis of 6 bacterial isolates representing different BOX-PCR fingerprint profiles are presented in Fig. 2, in addition to the reference bacterial strain *Lacticaseibacillus casei*. The 16S rRNA sequence of isolates 30, 58, and 65 showed 100% similarity to *Enterococcus faecalis*, isolate 57 was 100% similar to *Enterococcus faecium*, while isolate 68 showed 100% similarity to *Enterococcus hirae*. 16S rRNA sequences were deposited in the Gene Bank under the accession numbers from MN120883 to MN120887 in Table 3.

**Table 3:** Bacterial identification and accession numbers of isolates

Isolates No.	Identification	Gene bank closest hit		Accession number
30	*Enterococcus faecalis*	100.00%	MN749533.1	MN120883
57	*Enterococcus faecium*	100.00%	MN120884.1	MN120884
58	*Enterococcus faecalis*	100.00%	MN120885.1	MN120885
65	*Enterococcus faecalis*	100.00%	MN120886.1	MN120886
68	*Enterococcus hirae*	100.00%	MN629240.1	MN120887

### Optimization of fermentation process parameters for lactic acid production

To maximize LA production, the most active two strains *Enterococcus faecalis*-58 and *Enterococcus hirae*-68 were used to produce LA using mixture of whey permeate and salted cheese whey (1:1) as presented in Table 4.

**Table 4:** Effect of inoculum size, incubation temperature, and CaCO_3_ concentration on lactic acid production

Bacterial strains	Lactic acid (g/l) (mean ± SD)	Consumed sugar (g/l)	Yield (%)	Efficiency (%)	Lactic acid (g/l) (mean ± SD)	Consumed sugar (g/l)	Yield (%)	Efficiency (%)	Lactic acid (g/l) (mean ± SD)	Consumed sugar (g/l)	Yield (%)	Efficiency (%)	LSD at 0.05 for lactic acid production
Inoculum size	2%				3%				4%				0.326
*Enterococcus faecalis*-58	9.66 ± 0.34	24.1	40	48	9.85 ± 0.13	24.8	40	50	16.23 ± 0.23	28.3	57	57	
*Enterococcus hirae-*68	8.55 ± 0.26	21.9	39	44	8.69 ± 0.28	23.7	37	47	16.27 ± 0.27	27.6	59	55	
Incubation temperature	30 °C				37 °C				45 °C				0.277
*Enterococcus faecalis*-58	9.98 ± 0.18	25.7	39	51	16.23 ± 0.23	28.3	57	57	9.7 ± 0.2	25.1	39	50	
*Enterococcus hirae-*68	8.50 ± 0.12	24.5	35	49	16.27 ± 0.27	27.6	59	55	8.28 ± 0.28	23.3	36	47	
CaCO_3_ concentration	0%				0.5%								0.314
*Enterococcus faecalis*-58	10.9 ± 0.3	26.3	41	53	16.23 ± 0.23	28.3	57	57					
*Enterococcus hirae-*68	9.03 ± 0.08	25	36	50	16.27 ± 0.27	27.6	59	55					

Substrate: mixture of salted cheese whey and whey permeate in ratio (1:1) with Initial sugar concentration of 5%

### Inoculum size

To study the effect of inoculum size on LA production, different inocula sizes (2, 3, and 4 %, v/v) were added to the fermentation medium supplemented with SN nutrients and CaCO_3_ 0.5% at 37 °C for 36 h under static condition. LA production increased with increasing inoculum size up to 4% being 16.23 ± 0.23 and 16.27 ± 0.27 g/l with yields of 57 and 59% by *Ent. faecalis*-58 and *Ent. hirae-*68, respectively, LSD at 0.05 was 0.326.

### Incubation temperature

To determine the optimum temperature for LA production, the medium of salted whey and whey permeate mixture supplemented with SN, inoculum size (4%), CaCO_3_ (0.5%) under static condition was incubated at different temperatures 30, 37, and 45 °C. Incubation at 37 °C gave the highest LA production levels of 16.23 ± 0.23 and 16.27 ± 0.27 g/l and yields of 57 and 59% using *Ent. faecalis*-58 and *Ent. hirae*-68, respectively; LSD at 0.05 was 0.277.

### Addition of CaCO_3_

In the presence of two different CaCO_3_ concentrations (0% and 0.5%), the fermentation medium of (whey permeate and salted whey mixture (1:1)) supplemented with SN was inoculated by 4% inoculum size and incubated at 37 °C for 36 h under static condition. CaCO_3_ 0.5% concentration seemed better than CaCO_3_-free medium, where production of LA reached 16.23 ± 0.23 and 16.27 ± 0.27 g/l with yields of 57 and 59% for *Ent. faecalis*-58 and *Ent. hirae-*68, respectively; LSD at 0.05 was 0.314.

### Production of LA using immobilized cells

#### Immobilization of bacterial cells

Analytical growth of beads revealed that 1.6 × 10^9^ CFU/ml were immobilized in one gram beads of *Ent. faecalis*-58, *Ent. hirae*-68, dual of them and *L*. *casei* cells.

#### Effect of incubation period on LA production in salted cheese whey and whey permeate mixture (1:1) using immobilized cells and repeated batches fermentation

Repeated batches were used for LA production with immobilized cells of *Ent. faecalis*-58, *Ent. hirae*-68, mixture of them as well as *L. casei* under optimum conditions, which were determined based on batch fermentation results. Data represented in Table 5 showed that the maximum LA production and yield by *Ent. hirae*-68 were 36.13 ± 0.93 g/l and 89%, respectively, after 36 h of incubation period, while after 72 h of incubation, the maximum LA production and yield were 40.04 ± 0.54 g/l and 89%, respectively. After 96 h of incubation, the maximum LA production and yield reached 41 ± 0.4 g/l and 89%, respectively; LSD at 0.05 for lactic acid production was 1.22. In Table 6, using immobilized *Ent. faecalis*-58, the maximum LA production and yield were 36.95 ± 0.14 g/l and 81%, respectively, after 36 h of incubation period. After 72 h of incubation, the maximum LA production and yield reached 42.07 ± 0.1 g/l and 89%, respectively. After 96 h of incubation, the maximum LA production and yield were 42 ± 0.3 g/l and 89%, respectively; LSD at 0.05 for lactic acid production was 0.633. From the point of economic, 36 h was chosen to complete the next experiments.

**Table 5:** Effect of incubation period on lactic acid production using immobilized cells of *Enterococcus hirae-*68

Incubation period (h)	Number of runs	Lactic acid (g/l) (mean ± SD)	Consumed sugar (g/l)	Yield (%)	Efficiency (%)
24	(1–18) 18	8 ± 0.7–10 ± 0.8	13–14.8	62–68	26–30
36	(1) 1	13.5 ± 0.7	18.95	71	38
	(2–3) 2	24.41 ± 0.41–35.5 ± 0.5	31.18–42.22	78–84	62–84
	(4–8) 5	31.27 ± 0.47–34.1 ± 0.4	36.4–40	86–85	73–80
	(9–10) 2	28.15 ± 0.15–33.03 ± 0.43	33.28–38.16	85–87	67–76
	(11–13) 3	35.06 ± 0.26–36.13 ± 0.93	42.13–40.82	83–89	84–82
	(14–18) 5	30.36 ± 0.36–34.1 ± 0.7	35.48–39	86–87	71–78
72	(1–3) 3	34.1 ± 0.9–37.11 ± 0.91	39.03–42.38	87–88	78–85
	(4–6) 3	38.43 ± 0.43–39.5 ± 0.9	43.8–44.5	88–89	88–89
	(7–9) 3	36.47 ± 0.47–40.04 ± 0.54	41.69–45.2	87–89	83–90
	(10–12) 3	33.09 ± 0.19–35.96 ± 0.34	38.19–41.9	87–86	76–84
	(13–18) 6	25.3 ± 0.4–29.9 ± 0.9	34–37.9	74–79	68–76
96	(1–4) 4	37.12 ± 0.32–39 ± 0.8	42.29–44	88–89	85–88
	(5–9) 5	40.19 ± 0.44–41 ± 0.3	45.09–45.8	89–90	90–92
	(10–13) 4	36.07 ± 0.77–37 ± 0.8	41.15–42.5	88–87	82–85
	(14–16) 3	40.24 ± 0.36–41 ± 0.4	45.61–46	88–89	91–92
	(17–18) 2	29.95 ± 0.95–32.27 ± 0.27	39.2–40.89	76–79	78–82
LSD at 0.05		1.22			

Substrate: salted cheese whey and whey permeate mixture (1:1) with CaCO_3_ (0.5%), inoculum size (4%)

**Table 6:** Effect of incubation period on lactic acid production using immobilized cells of *Enterococcus faecalis*-58

Incubation period (h)	Number of runs	Lactic acid (g/l) (mean ± SD)	Consumed sugar (g/l)	Yield (%)	Efficiency (%)
24	(1–18) 18	7.97 ± 0.47–10 ± 0.4	13.5–14.8	59–68	27–30
36	(1) 1	16.43 ± 0.43	21.58	76	43
	(2–10) 9	29.38 ± 0.38–34.5 ± 0.4	35.45–42.59	83–81	71–85
	(11–12) 2	36.5 ± 0.3–36.95 ± 0.14	45.65–45.69	80–81	91–91
	(13–15) 3	30.62 ± 0.31–32.19 ± 0.19	35.79–37	85–87	72–74
	(16–18) 3	34.42 ± 0.3–36.1 ± 0.3	40.39–44.52	85–81	81–89
72	(1–5) 5	36.5 ± 0.4–39.49 ± 0.19	41.9–44.82	87–88	84–90
	(6–8) 3	39 ± 0.22–40.19 ± 0.19	44.11–45.33	88–89	88–91
	(9–10) 2	40.2 ± 0.2–42.07 ± 0.1	45.36–47.3	89–89	91–95
	(11–14) 4	34.59 ± 0.32–35.43 ± 0.23	44.85–41.78	77–88	90–84
	(15–18) 4	25.2 ± 0.2–31.75 ± 0.24	34.7–37.2	73–85	69–74
96	(1–9) 9	41.21 ± 0.21–42 ± 0.3	46.17–47	89–89	92–94
	(10–16) 7	38.48 ± 0.15–40.95 ± 0.36	43.66–45.91	88–89	87–92
	(17–18) 2	32.2 ± 0.2–33.38 ± 0.38	40.8–41.6	79–80	82–83
LSD at 0.05		0.633			

Substrate: salted cheese whey and whey permeate mixture (1:1) with CaCO_3_ (0.5%), inoculum size (4%)

In Table 7, using mixture of immobilized cells of *Ent. faecalis*-58 and *Ent. hirae-*68 strains, LA production and yield reached 35 ± 0.11 g/l and 84%, respectively, after 36 h of incubation period, but they were 38 ± 0.11 g/l and 85% after 72 h of incubation and 31 ± 0.08 g/l and 82% after 96 h of incubation; LSD at 0.05 for lactic acid production was 0.327. For using of *L*. *casei* in Table 8, after 36 h of incubation period, the maximum LA production and yield were 36 ± 0.11 g/l and 77% respectively, while after 72 h were 35 ± 0.22 g/l and 74% in addition to 40 ± 0.3 g/l and 83% after 96 h of incubation period; LSD at 0.05 for lactic acid production was 0.502. Although immobilized entrapped cells could be used during repetitive batch fermentations for more than 72 days (96 h incubation for 18 runs), increasing LA production by increasing of incubation period was not significant, so incubation for 36 h was deemed effective and economic.

**Table 7:** Effect of incubation period on lactic acid production using immobilized cells (*Enterococcus faecalis*-58 and *Enterococcus hirae-*68)

Incubation period (h)	Number of runs	Lactic acid (g/l) (mean ± SD)	Consumed sugar (g/l)	Yield (%)	Efficiency (%)
24	(1–18) 18	6.82 ± 0.12–8 ± 0.3	11.4–13.8	60–58	23–28
36	(1–3) 3	10.1 ± 0.1–11 ± 0.2	15.2–16.7	66–66	30–33
	(4–7) 4	16.3 ± 0.3–17 ± 0.4	21.2–22.8	77–75	42–46
	(8–10) 3	28 ± 0.4–30 ± 0.45	33.8–36.4	83–82	68–78
	(11–18) 8	34 ± 0.09–35 ± 0.11	39.2–41.5	87–84	78–83
72	(1–2) 2	12.88 ± 0.12–14 ± 0.39	17.92–20	72–70	36–40
	(3–9) 7	36.3 ± 0.3–37 ± 0.33	42.1–43.2	86–86	84–86
	(10–18) 9	36.82 ± 0.11–38 ± 0.11	42.9–44.5	86–85	86–89
96	(1–18) 18	29.03 ± 0.03–31 ± 0.08	34.1–37.6	85–82	68–75
LSD at 0.05		0.327			

Substrate: salted cheese whey and whey permeate mixture (1:1) with CaCO_3_ (0.5%), inoculum size (4%)

**Table 8:** Effect of incubation period on lactic acid production using immobilized cells of *Lacticaseibacillus casei*

Incubation period (h)	Number of runs	Lactic acid (g/l) (mean ± SD)	Consumed sugar (g/l)	Yield (%)	Efficiency (%)
24	(1–11) 11	14.93 ± 0.12–16 ± 0.52	27.7–29	54–55	55–58
	(12–18) 7	9.5 ± 0.1–10 ± 0.31	20–21.18	47.6–47	40–42
36	(1–3) 3	35.93 ± 0.05–36 ± 0.11	46.1–46.5	77.9–77	92–93
	(4–8) 5	26.9 ± 0.03–28 ± 0.07	41.2–43.1	65.4–65	82–86
	(9–12) 4	15.95 ± 0.05–17 ± 0.06	28.3–30.9	56.4–55	57–62
	(13–18) 6	15.1 ± 0.1–16 ± 0.3	27.3–29.4	55.3–54	55–59
72	(1–2) 2	28.2 ± 0.2–29 ± 0.08	42.5–44.3	66.3–65	85–89
	(3–6) 4	34.85 ± 0.65–35 ± 0.22	46–47.1	75.7–74	92–94
	(7–12) 6	34.15 ± 0.15–35 ± 0.41	45.2–47	75.5–74	90–94
	(13–18) 6	29.32 ± 0.32–30 ± 0.09	43.1–44.6	68–67	86–89
96	(1–18) 18	39.2 ± 0.2–40 ± 0.3	45.5–48.3	86–83	91–97
LSD at 0.05		0.502			

Substrate: salted cheese whey and whey permeate mixture (1:1) with CaCO_3_ (0.5%), inoculum size (4%)

### Effect of sugar concentration on LA production from salted cheese whey and whey permeate mixture using immobilized cells and repeated batch fermentation

**Fig. 4:**
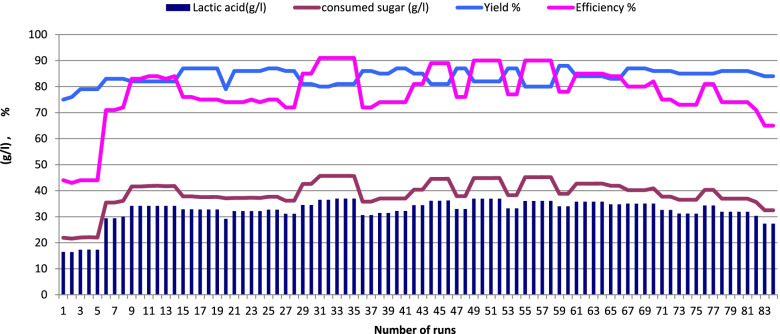
Lactic acid production from salted cheese whey and whey permeate mixture (1:1, contained 5% sugar) using immobilized cells of *Enterococcus faecalis*-58

**Fig. 5:**
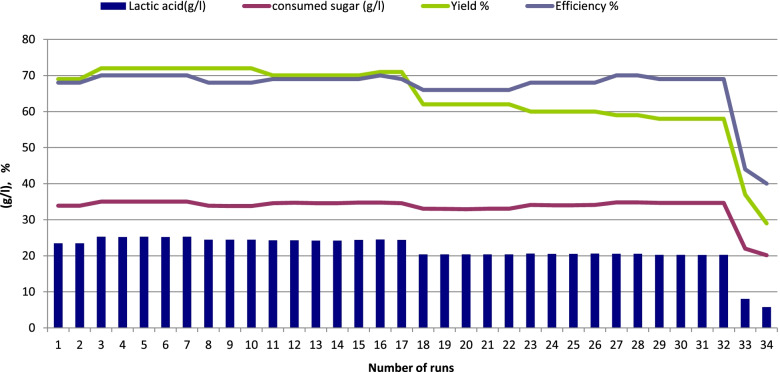
Lactic acid production from salted cheese whey and whey permeate mixture (1:1, contained 10% sugar) using immobilized cells of *Enterococcus faecalis*-58

**Fig. 6:**
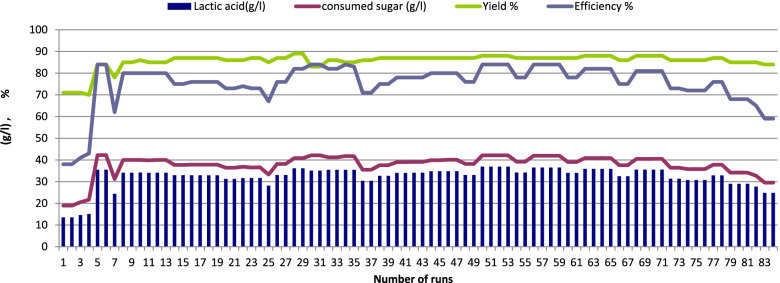
Lactic acid production from salted cheese whey and whey permeate mixture (1:1, contained 5% sugar) using immobilized cells of *Enterococcushirae*-68

**Fig. 7:**
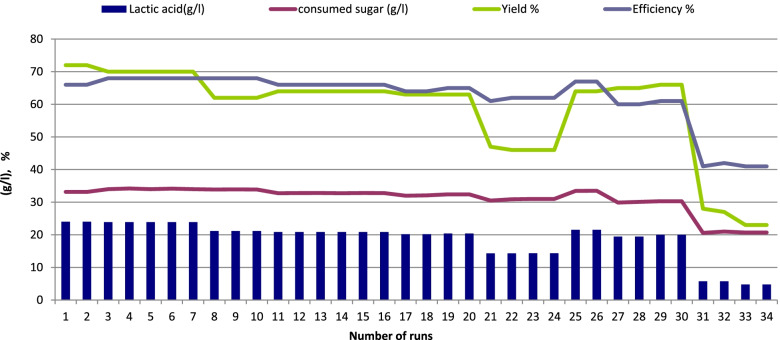
Lactic acid production from salted cheese whey and whey permeate mixture (1:1, contained 10% sugar) using immobilized cells of *Enterococcus hirae*-68

Two different sugar concentrations were used (5 and 10% sucrose) to examine their effects on LA production by immobilized cells of *Enterococcus faecalis*-58 and *Enterococcus hirae-*68 at 37 °C for 126 days representing 84 runs (each run 36 h). As shown in Figs. 4 and 5 (*Enterococcus faecalis*-58), results indicated that 5% sugar was better than 10% sugar where LA production reached 36.95 g/l (LSD at 0.05 = 0.244) with yield of 81% using 5% sugar and decreased to 25.3 g/l (LSD at 0.05 = 0.315) with yield of 72 %, respectively as a result of sugar concentration increased to 10%. For immobilized cells of *Enterococcus hirae-*68 (Figs. 6 and 7), it was recorded that 5% sugar was effective than 10% sugar, where LA production reached 36.91 g/l (LSD at 0.05 = 0.258) with yield of 88% at 5% sugar which was higher than in 10% sugar which reached 24.03 g/l (LSD at 0.05 = 0.408) with yield of 72%, respectively.

## Discussion

Cheese whey can be used as a substrate for production of lactic acid by microbial fermentation to manage and reduce environmental pollution. Study of [25] found that *Lacticaseibacillus casei* produced 33.73 g/l of LA during 36 h using fermentation of cheese whey at 37 °C while [26] recorded maximum LA production of 2.53 g/l at 42 °C. Another study recorded almost a similar level of LA (16 g/l) by immobilized *L*. *casei* in batch culture [27]. Also, salted cheese whey could be used for production of carotenoid using different strains of yeasts [28]. Our results are in agreement with [27] who reported that the highest concentration of LA (16 g/l) in batch fermentation was attained at 28 °C and pH 5.5 after 5 days of incubation by immobilized *L*. *casei* and the highest concentration of LA (14.8 g/l) in continuous fermentation was attained at 32 °C and pH 5 after 5 days of incubation. LAB could be used for bio-production of LA from wastes of dairy industries. In this context, [29] isolated *Enterococcus faecalis* CBRD01 from soil and used it for LA production. Also [30] isolated *Enterococcus hirae* and used it for LA production. In the same direction, [31] could use new isolated strain of *Enterococcus faecium* for production of lactic acid.

Panesar [25] scored maximum LA production of 33.72 g/l with 2–4% inoculum size of bacterial culture. Also, [26] observed maximum LA production of 2.53 g/l with 4% inoculum of bacterial culture. Other studies, [26] observed maximum LA production of 2.53 g/l at 42 °C, while [25] observed maximum LA production of 33.72 g/l at 37 °C. Also, [5] found that the fastest and highest LA production from whey using *L*. *casei* was obtained at 37 °C. Most of the lactic acid-producing enterococci described in the literature show extraordinary production of lactic acid at the temperature ranged between 30 and 43 °C. In this context, [31] found that 40 °C was the optimal temperature for LA production by *Ent. faecium*, [32] reported that 38 °C was the optimal temperature for LA production by *Ent. faecalis* RKY1, and [33] found that 30 °C was the optimal temperature for the production of lactic acid from sago starch using *Ent. faecalis*. It is important to maintain the operating temperature at the optimal level because it affects the growth rate, enzymes activity, biochemical reactions, as well as substrate consumption rate and LA production efficiency [34]. Researchers reported that addition of calcium carbonate was important to neutralize the acid produced during fermentation using LAB [5, 25]. Results of the present study (Table 4) indicated that the optimum conditions for LA production were 37 °C, 4% inoculum size, and 0.5% CaCO_3_ concentration. Also, [35] reported that, production of lactic acid from mixture of salted whey and whey permeate (1:1) reached 27–38 g/l with efficiency ranged between 60 and 80% using *L*. *casei* and *L*. *rhamnosus* B-445 strains under the conditions of 5% sugar, 3% salt, and 0.5% calcium carbonate during static state fermentation at 37 °C. Hitha [36] reported that LA production using immobilized cells of LAB in sodium alginate beads during fermentation of cheese whey increased to 109 g/l, while it was 60 g/l using free cells. El-Gizawy [37] encapsulated *Lactobacillus delbrueckii subsp. bulgaricus* with sodium alginate for improving quality of Kareish cheese. Abdel-Rahman [30] obtained maximum LA production of 19.6 g/l using 20 g/l glucose in batch fermentation by *Ent. hirae*.

LAB such as *Lactobacillus* and *Lactococcus* with fed-batch or repeated batch fermentations were used to produce lactic acid [38]. Moreover, [39] could obtain five isolates of lactic acid producing bacteria from cheese sample which were identified genetically using 16S rRNA as *Lacticaseibacillus casei* MT682513, *Enterococcus camelliae* MT682510, *Enterococcus faecalis* MT682509, *Enterococcus lactis* MT682511, and *Wissella paramesenteroids* MT682512. *Lacticaseibacillus casei* could produce the highest production of lactic acid (44.9 g/l) using whey permeate and small scale batch fermentation without any supplementation. The maximum lactic acid productions were obtained at 30–37 °C for all isolates. Likewise, [40] found that wild strain of *Lacticaseibacillus casei* BL23 could produce 2.89 g/l of l-lactate after 95 h during batch fermentation of cheese whey, l-lactate production increased to 8.13 g/l with the addition of 0.5% yeast extract as a source of nitrogen.

Our results are in agreement with [20] who reported that the highest LA production was 35 g/l when they used *Streptococcus thermophilus* and *Lactobacillus delbrueckii subsp. bulgaricus* co-immobilized in high viscosity beads (1% w/v alginate) hardened in 0.1 M CaCl_2_ which was lower than the maximum concentration achieved in the present study. Consistently, immobilized cells of *Lactiplantibacillus plantarum* in continuous fermentation could produce lactic acid from cheese whey with titer (33.8 g/l), yield (88%), and productivity (11.3 g/l h.) [41]. Consequently, [42] deduced that cell immobilization alters cell membrane due to increasing the permeability and LA production [43]. found that repeated batch fermentation using *Lacticaseibacillus casei* in alginate entrapped cells decreased the fermentation time by half with volumetric productivity of 0.625 g/l h compared to 0.375 g/l h using free cells fermentation; therefore, immobilized cells could be used in repetitive batch fermentation for more than 40 days, where the maximum productivity in the present study was 1.03 g/l h. In the same direction, fermentation of glucose using *L*. *casei* in immobilized form on gluten pellets needs shorter time (18 h) to produce higher LA (42 g/l) compared to free cells [44]. Also, immobilized cells of *Lactiplantibacillus pentosus* had higher heat stability and higher LA production rate from fructose [45]. Moreover, *Lactiplantibacillus plantarum* could use industrial wastes such as whey as substrates for LA production [46]. In comparison with batch or fed batch culture, repeated batch operation has proved to have several advantages in increasing LA productivity besides saving the time and labor work [30]. Luongo [47] obtained maximum LA production concentration of 20.1 g/l and maximum yield of 37% using repeated batch fermentation of cheese whey for LA production during semi-continuous fermentation by mixed cultures.

High concentration of sugar causes osmotic stress and long lag phase of LAB resulting in low sugar consumption and LA production, fed-batch fermentation can be used to reduce substrate inhibition to maximize LA production [38]. Moreover, [47] obtained maximum LA production using sugar concentration of 20.1 g/l with maximum yield of 37 % using repeated-batch fermentation of cheese whey during semi-continuous fermentation by mixed cultures. Abdel-Rahman [30] studied LA production in repeated fermentation process for ten repeated runs; the authors reported that LA productivity increased when the total of ten repeated runs were carried out using 60 g/l glucose but the productivity decreased by increasing glucose concentration to 100 g/l.

## Conclusion

Salted cheese whey can be used for lactic acid production in mixture with whey permeate using immobilized cells of the promising bacterial strains *Enterococcus faecalis* and *Enterococcus hirae* using static state fermentation under the optimum conditions of (4% inoculum size, in mixture contained 5% sucrose and 0.5% calcium carbonate, with incubation at 37 °C). Sodium alginate immobilized entrapped cells exhibited good mechanical strength during fermentation and could be used in repetitive batch cultures for more than 126 days.

## Data Availability

The authors declare that all data supporting the findings of this study are included within the article and its supplementary information file.

## Abbreviations

LAB: Lactic acid bacteria
LA: Lactic acid
*L.*: *Lacticaseibacillus*
*Ent.*: *Enterococcus*
*Lac.*: *Lactococcus*
CFU: Colony-forming unit
DNA: Deoxyribonucleic acid
RNA: Ribonucleic acid
rRNA: Ribosomal ribonucleic acid
PCR: Polymerase chain reaction
DNS: Dinitrosalicylic acid
NCBI: National Center for Biotechnology Information
HPLC: High-performance liquid chromatograph
SD: Standard deviation
LSD: Least significant difference
